# Legal Responsibilities

**Published:** 1856-10

**Authors:** 


*Legal Responsibilities.*—Judge Minot, of Pennsylvania, has laid down the following rules of law, as applicable to physicians:

1. The medical man engages that he possesses a reasonable degree of skill, such as is ordinarily possessed by practitioners generally.

2. He engages to exercise that skill with reasonable care and diligence.

3. He engages to exercise his best judgment, *but is not responsible for mistake of judgment.* Beyond this the defendant is not responsible. The patient himself is responsible for all else; if he desires the highest degree of skill and care, he must secure it himself.

4. It is a rule of law, that a medical practitioner never insures the result. These are received in general as sound views, and such will govern every enlightened court. There could scarcely be a greater absurdity than to require physicians and surgeons to insure the result, when they can in no case control all parts of the treatment. Few serious cases are carried through a single day, and many not a single hour, without a violation of instructions on the part of nurses and attendants.—*Cincinnati Med. Observer.*

